# Heterogeneity of Cell Surface Glutamate and GABA Receptor Expression in Shank and CNTN4 Autism Mouse Models

**DOI:** 10.3389/fnmol.2018.00212

**Published:** 2018-06-19

**Authors:** Christopher Heise, Jonathan M. Preuss, Jan C. Schroeder, Chiara R. Battaglia, Jonas Kolibius, Rebecca Schmid, Michael R. Kreutz, Martien J. H. Kas, J. Peter H. Burbach, Tobias M. Boeckers

**Affiliations:** ^1^Institute for Anatomy and Cell Biology, Ulm University, Ulm, Germany; ^2^RG Neuroplasticity, Leibniz Institute for Neurobiology, Magdeburg, Germany; ^3^Groningen Institute for Evolutionary Life Sciences, University of Groningen, Groningen, Netherlands; ^4^Department of Translational Neuroscience, Brain Center Rudolf Magnus, University Medical Center Utrecht, Utrecht, Netherlands

**Keywords:** autism spectrum disorder, autism mouse models, Shank2, Shank3, Cntn4, synapse, cell surface receptors, biotinylation assay

## Abstract

Autism spectrum disorder (ASD) refers to a large set of neurodevelopmental disorders, which have in common both repetitive behavior and abnormalities in social interactions and communication. Interestingly, most forms of ASD have a strong genetic contribution. However, the molecular underpinnings of this disorder remain elusive. The *SHANK3* gene (and to a lesser degree *SHANK2*) which encode for the postsynaptic density (PSD) proteins SHANK3/SHANK2 and the *CONTACTIN 4* gene which encodes for the neuronal glycoprotein CONTACTIN4 (CNTN4) exhibit mutated variants which are associated with ASD. Like many of the other genes associated with ASD, both *SHANKs* and *CNTN4* affect synapse formation and function and are therefore related to the proper development and signaling capability of excitatory and inhibitory neuronal networks in the adult mammal brain. In this study, we used mutant/knock-out mice of Shank2 (*Shank2*^−/−^), Shank3 (*Shank3*αβ^−/−^), and Cntn4 (*Cntn4*^−/−^) as ASD-models to explore whether these mice share a molecular signature in glutamatergic and GABAergic synaptic transmission in ASD-related brain regions. Using a biotinylation assay and subsequent western blotting we focused our analysis on cell surface expression of several ionotropic glutamate and GABA receptor subunits: GluA1, GluA2, and GluN1 were analyzed for excitatory synaptic transmission, and the α1 subunit of the GABA_A_ receptor was analyzed for inhibitory synaptic transmission. We found that both *Shank2*^−/−^ and *Shank3*αβ^−/−^ mice exhibit reduced levels of several cell surface glutamate receptors in the analyzed brain regions—especially in the striatum and thalamus—when compared to wildtype controls. Interestingly, even though *Cntn4*^−/−^ mice also show reduced levels of some cell surface glutamate receptors in the cortex and hippocampus, increased levels of cell surface glutamate receptors were found in the striatum. Moreover, *Cntn4*^−/−^ mice do not only show brain region-specific alterations in cell surface glutamate receptors but also a downregulation of cell surface GABA receptors in several of the analyzed brain regions. The results of this study suggest that even though mutations in defined genes can be associated with ASD this does not necessarily result in a common molecular phenotype in surface expression of glutamatergic and GABAergic receptor subunits in defined brain regions.

## Introduction

The term autism spectrum disorder (ASD) refers to a spectrum of heterogeneous developmental disorders, which share two striking behavioral phenotypes: patients exhibit repetitive, stereotypic behavior and they also show impaired social communication and behavior. Importantly, ASD has a strong genetic component: if one sibling has ASD, the other has a 25× higher risk of developing ASD as compared to the general population; the concordance rate of monozygotic twins is 70%–90% compared with 0%–10% in dizygotic twins and males have a four times higher risk of developing ASD as compared to females (Moessner et al., 2007; Fassio et al., 2011). ASD also appears to be linked with changes in excitatory and/or inhibitory network activity as up to one-third of the ASD patients also suffer from epilepsy (Rapin, 1997; Tuchman and Rapin, 2002). This may be related to the fact that several of the genes related to ASD are synaptic proteins or proteins, which have synaptic functions (Mullins et al., 2016). It should be noted that the majority of ASD cases are due to genetic variations in a multitude of genes and only a minority of cases can be linked to monogenetic variants of genes, such as variations in the *SHANK3*, *SHANK2* and *CONTACTIN4* (CNTN4) genes (Pinto et al., 2010; Leblond et al., 2014; Mullins et al., 2016). But even in the cases where ASD can be associated with a variant of a single gene, our knowledge of how these genes are linked to this disorder needs to be improved and will likely better our understanding not only of those specific ASD cases but also of mechanisms underlying the entire range of ASD.

The *SHANK* gene family (*SHANK1*, *SHANK2* and *SHANK3)* encodes for postsynaptic density (PSD) associated proteins at the excitatory synapse that act as scaffolds and interconnect neurotransmitter receptors and cell adhesion molecules—both by direct and indirect interactions with a large group of other PSD associated proteins (Boeckers et al., 2002; Grabrucker et al., 2011; Jiang and Ehlers, 2013). Several research groups have demonstrated the importance of Shanks for the proper functioning of the excitatory synapse and excitatory synaptic transmission (Sala et al., 2001; Peca et al., 2011; Schmeisser et al., 2012; Vicidomini et al., 2017) and Shank3 haploinsufficiency has not only been linked to ASD but also to schizophrenia and neuropsychiatric symptoms in the Phelan-McDermid syndrome (Guilmatre et al., 2014). CNTN4/BIG-2, an neuronal glycoprotein, which belongs to a subfamily of the immunoglobulin superfamily of cell adhesion molecules (Oguro-Ando et al., 2017) is known to guide axons during development. This function has been studied in the development of the olfactory and visual neural circuit (Kaneko-Goto et al., 2008; Osterhout et al., 2015). However, the precise role of CNTN4 in establishing neural networks and synaptic contacts remains to be clarified.

Unraveling the molecular and network abnormalities in the brain that cause ASD has proven to be a difficult task and has been carried out in a multidisciplinary fashion (Baudouin et al., 2012; Bourgeron, 2015; Mullins et al., 2016). Part of this research has focused on comparing ASD related genes with respect to function, subcellular localization and the expression pattern in the brain. Other lines of research took advantage of mouse models for monogenetic ASD to unravel novel ASD specific phenotypes at the electrophysiological and biochemical level. We followed the latter approach and utilized mutant/knock-out mice of Shank2 (*Shank2*^−/−^), Shank3 (*Shank3*αβ^−/−^) and Cntn4 (*Cntn4*^−/−^) to see whether these mice share common cell surface expression patterns of glutamate and GABA receptors in ASD related brain areas: cortex, striatum, hippocampus, thalamus and cerebellum (Ameis et al., 2011; Peca et al., 2011; Becker and Stoodley, 2013; Schuetze et al., 2016). To this end, a cell surface biotinylation protocol was established on acute coronal mouse brain slices containing the aforementioned brain regions, followed by a western blot analysis for several ionotropic glutamate and GABA receptor subunits. Antibodies directed against GluA1, GluA2 and GluN1 were used for the analysis of excitatory synaptic transmission, and antibodies raised against the α1 subunit of the GABA_A_ receptor were used for the analysis of inhibitory synaptic transmission.

Here, we report that *Shank2*^−/−^ and *Shank3*αβ^−/−^ mice have reduced expression levels of several cell surface glutamate receptors which is most evident in the striatum and thalamus. *Shank2*^−/−^ also present lower expression levels in the cortex and cerebellum. In contrast, *Cntn4*^−/−^ mice have lower expression levels of cell surface glutamate receptors in the cortex and hippocampus but increased expression levels of cell surface glutamate receptors in the striatum. In addition, *Cntn4*^−/−^ mice differ from *Shank2*^−/−^ and *Shank3*αβ^−/−^ mice since they not only show brain region specific changes in cell surface glutamate receptors but also a downregulation of cell surface GABA receptors in several brain regions. Taken together, the results of this study indicate that monogenetic variants in genes associated with ASD may not necessarily have a common molecular phenotype at the level of excitatory and inhibitory signaling components such as ionotropic glutamate and GABA receptors. This raises the question whether variants in genes related to ASD may have commonalities at other levels of information processing, such as at the level of neuronal networks, which has become a relevant aspect in ASD research (Baudouin et al., 2012; Zikopoulos and Barbas, 2013; Bourgeron, 2015; Mullins et al., 2016).

## Materials and Methods

### Animal Ethics Statement

*Shank2*^−/−^, *Shank3*αβ^−/−^ and *Cntn4*^−/−^ mice were previously described (Kaneko-Goto et al., 2008; Schmeisser et al., 2012). All mice were kept in specific pathogen-free animal facilities and all animal experiments in this study were performed based on the guidelines for the welfare of experimental animals issued by the Federal Government of Germany and by the local ethics committee (Ulm University), ID Number: 0.103.

### Primary Antibodies

Primary antibodies used for western blotting were diluted 1:500 (except for actin which was diluted 1:100,000). The following primary antibodies were purchased from commercial suppliers: Actin (Sigma-Aldrich Cat# A2228 RRID:AB_476697), NR1/GluN1 (Sigma-Aldrich Cat# G8913 RRID:AB_259978), GluA1 (SynapticSystems Cat# 182011 RRID:AB_2113443), GluA2 (SynapticSystems Cat# 182111 RRID:AB_10645888), GABA_A_Rα1 (NeuroMab Cat# N95/35 RRID:AB_2108811), pERK (Cell Signalling Cat# 9101 RRID:AB_2297442), mGluR5 (Millipore Cat# AB5675 RRID:AB_2295173).

### Secondary Antibodies

Secondary antibodies used for western blotting were HRP-conjugated (Dako, Glostrup, Denmark, dilution 1:1000).

### Slice Preparation

The slice preparation protocol was derived from studies implementing classical electrophysiological recordings of acute brain slices (Mathis et al., 2011; Whitehead et al., 2013; Heise et al., 2017). Briefly, adult male mice aged 3–6 months (one *Shank2*^−/−^, *Shank3*αβ^−/−^, or *Cntn4*^−/−^ and a corresponding wildtype control mouse) were sacrificed and brains were extracted on ice in a petri dish filled with ice cold artificial cerebrospinal fluid (ACSF; NaCl, 120 mM; KCl, 2.5 mM; NaH_2_PO_4_, 1.25 mM; NaHCO_3_, 22 mM; Glucose, 10 mM; MgSO_4_, 2 mM; pH 7.4; oxygenation with 95% O_2_/5% CO_2_). Then, 300 μm coronal slices were made using a vibratome (Thermo scientific, Vibratom Microm HM 650V; “cutting frequency” = 100, “speed” = 10) beginning rostrally at Bregma 3.20 mm. Slices containing striatum and anterior cortex portions were gathered between Bregma 1.50 mm and Bregma 0.00 mm. Slices containing hippocampus, thalamus and posterior cortex portions were gathered between Bregma −1.00 mm and Bregma −3.00 mm. Lastly, slices containing cerebellum were gathered between Bregma −5.80 mm and Bregma −7.80 mm. After slicing, sections were immediately transferred to a 2 l glass beaker filled with ice cold ACSF (oxygenation with 95% O_2_/5% CO_2_) until all slices were gathered and cell surface biotinylation (see below) was carried out. View Supplementary Figure S2,1 for a visual impression of the aforementioned slices.

### Cell Surface Biotinylation

Cell surface biotinylation and NeutrAvidin pull-down was carried out with slight modifications of previously published work (Whitehead et al., 2013). Briefly, slices were transferred from the 2 l beaker (see “Slice Preparation” section above) to 6-well plates where they were incubated with ice cold Sulfo-NHS-SS-Biotin (Thermo #21331; 1 mg/ml solubilized in ACSF) on a horizontal shaker (Heidolph, Unimax 1010; around 90 rotations per minute (RPM)) for 45 min at 4°C. Then, several washing steps followed to stop the biotinylation reaction, each lasting 5 min on the horizontal shaker at 4°C: two washes with ice cold 10 mM glycine (solubilized in ACSF) and a final wash with ice cold TBS (150 mM NaCl, 50 mM Tris HCl, pH 7.4).

### Extraction of Brain Regions

Brain regions of interest (cortex, striatum, hippocampus, thalamus and cerebellum) were dissected from the various coronal brain slices using small forceps (Fine Science Tools, Heidelberg, Germany) under an Olympus SZ40 stereoscope. Anterior and posterior cortex sections (see above) were pooled.

### Lysis, NeutrAvidin Pull-Down, Input (Total) and Biotinylated Fraction (Surface)

Extracted, biotinylated brain regions (see above) were collected in 1.5 ml Eppendorf tubes containing 600 μl of lysis buffer (25 mM Tris (pH 7.6), 150 mM NaCl, 1% TritonTM X-100, 0.5% sodium deoxycholate, 0.1% sodium dodecyl sulfate (SDS), 2 mM NaF, 1 mM EDTA and a cocktail of protease inhibitors (Roche), diluted in dH_2_O). Tissue was then lysed on ice using a potter and centrifuged at 12000 *g* (Eppendorf; 5430R) for 15 min at 4°C to remove nuclei and cellular debris. Supernatant was transferred to fresh, precooled 1.5 ml Eppendorf tubes. Samples were then analyzed by Bradford assay to assess protein concentration. Samples were adjusted to 1 μg/μl using lysis buffer and 2× 25 μl were taken from each sample as input controls (5%) and stored at 4°C until later use (see below). Five-hundred microliter of 1 μg/μl sample were transferred to a new precooled 1.5 ml Eppendorf tube and 100 μl of 50% Neutravidin agarose resin (Thermo: #29200) slurry were added and incubated overnight on a test-tube-rotator (Shijders, 34528) at 4°C. The next day, several washing steps followed: 3× washes with ice cold lysis buffer and 2× washes with ice cold TBS (cocktail of protease inhibitors). Between each wash samples were put on the test-tube-rotator at 4°C for 30 min, followed by a centrifugation step at 1000 *g* to separate the Neutravidin agarose matrix and the attached biotinylated proteins from the supernatant. After the last washing step, proteins were eluted from the matrix with 60 μl of 4× loading dye (200 mM Tris-HCL, pH 6.8, 200 mM DTT, 4%SDS, 4 mM EDTA, 40% glycerol, 0.02% bromophenolblue), yielding the biotinylated fraction with biotinylated cell-surface-bound proteins, henceforth referred to as the surface fraction. At this point, input samples from the previous day were also diluted with 4× loading dye, yielding the input controls which contain both biotinylated and non-biotinylated proteins, henceforth referred to as the total protein fraction. Finally, samples were boiled for 5 min at 95°C, put directly on ice, centrifuged at 12,000 *g*, and saved for subsequent Western Blot analysis at −20°C.

### Western Blot Analysis

For western blotting, one input control (5%) and half of the biotin fraction of the wildtype mouse and the corresponding *Shank2*^−/−^, *Shank3*αβ^−/−^, or *Cntn4*^−/−^ mouse were loaded on one gel to compare the signal intensities of the fractions as faithfully as possible. Per experimental round, a second gel with the same loading was carried out to increase the amount of different antibodies that could be used per round. Western blot analysis was carried out according to standard protocols (Laemmli, 1970). HRP-conjugated secondary antibodies were used together with the SuperSignal detection system (Thermo Scientific) to visualize protein bands.

### Quantification of Protein Band Signal Intensity and Data Analysis

Digital files from the SuperSignal detection system were analyzed with the open source program ImageJ (US National Institutes of Health). Average band intensities of the signal of interest were calculated, adjusting for background noise. Then, the average band intensity of each surface fraction was divided by the corresponding average band intensity in the total fraction, yielding the “surface/total ratio.” After this, data was normalized to the average “surface/total ratio” of the wildtype control and the mutant mouse model of the experimental round, yielding the “normalized surface/total ratio.” In order to easily interpret changes between wildtype and mutant mice, the wildtype average was then set to 1 by dividing data by the mean of the wildtype control across experimental rounds. Statistical analysis was carried out using a student’s *t*-test. For total protein analysis, average band intensities of the signal of interest in the total fraction were normalized by actin and the wildtype average was then set to 1 as described above.

### Neuronal Stimulation Protocol

For the experiment pertaining to neuronal stimulation using “high potassium,” coronal slices containing the hippocampus were initially gathered in two 2 l glass beakers (one for the control and one for the experimental group) containing ice cold ACSF as described above. After 15 min, the ACSF in the glass beakers was exchanged quickly for an ice cold ACSF without MgSO_4_ but with 2 mM CaCl_2_ instead (all other buffer components were equal and again oxygenation took place with 95% O_2_/5% CO_2_; incubation for 30 min). Then, the temperature was raised 1°C/min to a final temperature of nearly 37°C. As soon as the final temperature was reached, a 4 M KCl stock (diluted in dH_2_O) was added to the ACSF of the experimental group to yield a final concentration of 50 mM KCl (“high potassium”). For the control group, no KCl but instead the corresponding volume of dH_2_O was added. After 5 min, slices were transferred to new 2 l glass beakers containing ice cold ACSF without MgSO_4_ but with 2 mM CaCl_2_ (again oxygenation took place with 95% O_2_/5% CO_2_). Then, cell surface biotinylation, extraction of the hippocampus, tissue lysis, NeutrAvidin pull-down and western blot analysis took place as described above (however, every buffer in downstream applications—e.g., the lysis buffer—contained 2 mM CaCl_2_).

## Results

### The Implemented Experimental Design Yields Clean Cell Surface Fractions and Acute Slices With Intact Intracellular Signaling Properties

**Figure 1:**
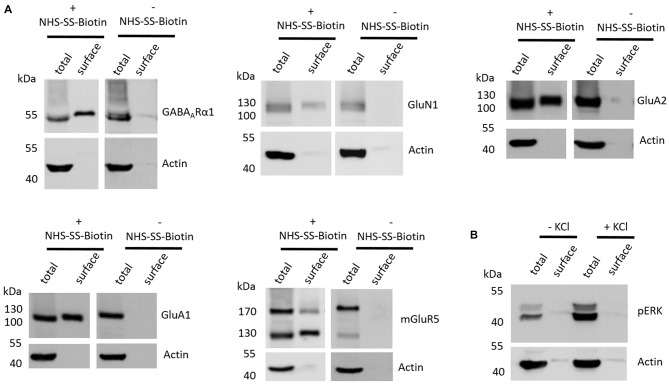
Various controls of the cell surface biotinylation procedure confirm a properly functioning experimental design yielding acute coronal slices with physiological signaling properties. **(A)** Representative western blots (WBs) showing band intensities of hippocampal total protein and cell surface sample with or without prior cell surface biotinylation reveal that cell surface samples are almost devoid of typical intracellular proteins and that biotinylation is necessary for enrichment of proteins in the cell surface sample. The cell surface biotinylation protocol either was (+NHS-SS-Biotin) or was not (−NHS-SS-Biotin) carried out on acute coronal wildtype slices containing the hippocampus prior to dissection and lysis of hippocampal tissue from the slice. Total protein samples (total) and samples containing biotinylated cell surface proteins (surface) were analyzed by WB for GABA_A_Rα1, GluN1, GluA2, GluA1, mGluR5 and actin. **(B)** Representative WBs showing band intensities of hippocampal total protein and cell surface sample with or without “high potassium” stimulation prior to cell surface biotinylation reveal that total protein samples exhibit an increased phospho ERK signal intensity after “high potassium” stimulation relative to control samples. Neuronal stimulation of acute coronal wildtype slices by 50 mM KCl for 5 min at 37°C either was (+KCl) or was not (−KCl) performed before cell surface biotinylation and subsequent dissection and lysis of hippocampal tissue from the slice. Total protein samples (total) and samples containing biotinylated cell surface proteins (surface) were analyzed by WB for pERK and actin.

The first aim of this study was to test whether the employed cell surface biotinylation protocol for acute coronal brain slices yields relatively pure cell surface fractions and whether the cell surface biotinylation is really a prerequisite for receptor accumulation in the cell surface fraction, as would be expected. For this analysis, we utilized hippocampal tissue extracted from slices between Bregma −1.00 mm and Bregma −3.00 mm (see “Materials and Methods” section and Supplementary Figure S1) which expresses high levels of ionotropic glutamate receptors (Schwenk et al., 2014). To test for the purity of the cell surface fraction we carried out a western blot analysis with actin as an abundant intracellular protein and found that actin is present in the total protein fraction which contains non-biotinylated and biotinylated proteins but is almost absent in cell surface fractions (Figure 1A). To test whether the cell surface biotinylation protocol is necessary to accumulate glutamate and GABA receptors in the cell surface fraction, we carried out our experimental procedure either with (+) or without (−) the use of NHS-SS-Biotin and found that, as expected, NHS-SS-Biotin is needed for the accumulation of GABA_A_Rα1, GluN1, GluA2, GluA1 and mGluR5 in the surface fraction (Figure 1A).

Lastly, we wanted to test whether our slice preparation and subsequent experimental procedures yield acute coronal brain slices with intact physiological properties. As an indication for the ability of the slices to react in a physiologically relevant manner we chose to stimulate our slices with “high potassium” (extracellular KCl concentration of 50 mM for 5 min at 37°C), which is a classical protocol to stimulate neurons *in vitro* and *ex vivo* and is related to an increase in ERK phosphorylation and various associated neuronal processes such as long-term potentiation (LTP). For this analysis, we again utilized hippocampal tissue extracted from slices between Bregma −1.00 mm and Bregma −3.00 mm since many studies utilizing this high potassium protocol to induce neuronal stimulation and ERK phosphorylation took advantage of hippocampal neurons (Sala et al., 2000; Lundquist and Dudek, 2006). We found that stimulating our slices before cell surface biotinylation, indeed, leads to an increase of ERK phosphorylation relative to non-stimulated controls, yielding increased levels of phospho-ERK running at 42 and 44 kDa in the sodium dodecyl sulfate polyacrylamide gel electrophoresis (SDS-PAGE; Figure 1B).

### *Shank2*^−/−^ and *Shank3*αβ^−/−^ Mice Exhibit Reduced Cell Surface Glutamate Receptor Levels in the Striatum and Other ASD Related Brain Regions

We started our investigation of cell surface glutamate and GABA receptor expression levels in ASD mouse models by applying the cell surface biotinylation protocol to acute slices from *Shank2*^−/−^ and *Shank3*αβ^−/−^ mice and comparing them to wildtype controls. After biotinylation, the following ASD related brain regions were dissected from the slices, lysed and analyzed by western blot: cortex, striatum, hippocampus, thalamus and cerebellum (Supplementary Figure S1). Immunodetection was carried out with antibodies directed against GABA_A_Rα1, GluA2, GluN1, GluA1 and mGluR5 and revealed that signal intensities in the cell surface fractions were high enough for quantification in most brain regions, whereas quantification of mGluR5 was limited to the hippocampus (Supplementary Figure S2,1).

**Figure 2:**
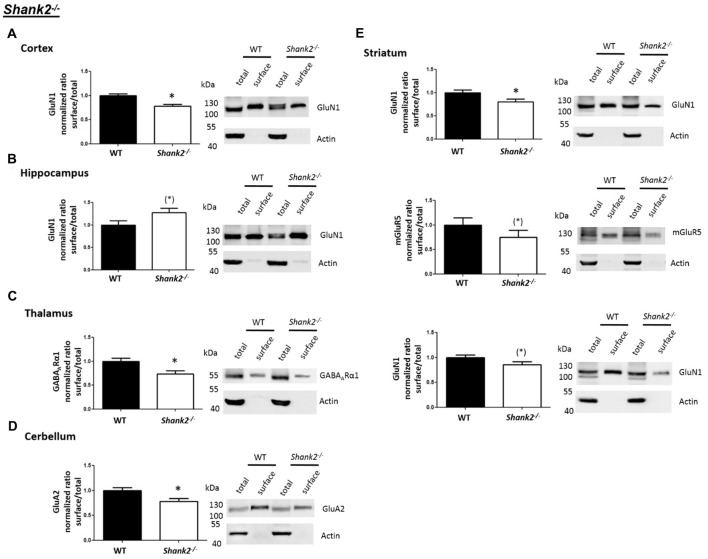
Analysis of cell surface glutamate and GABA receptor subunits in autism spectrum disorder (ASD) related brain regions of *Shank2*^−/−^ mice. **(A–E)** Representative WBs showing band intensities of total protein and cell surface sample in cortex **(A)**, striatum **(B)**, hippocampus **(C)**, thalamus **(D)** and cerebellum **(E)** of wildtype and *Shank2*^−/−^ mice reveal a downregulation of cell surface glutamate receptor subunits in *Shank2*^−/−^ mice. Immunodetections were carried out with antibodies directed against GABA_A_Rα1, GluA2, GluN1, GluA1, mGluR5 and actin. For GABA_A_Rα1, GluA2, and actin all brain regions were analyzed. For GluN1 all brain regions except the cerebellum were analyzed. For GluA1 all brain regions except the thalamus and cerebellum were analyzed. For mGluR5 only the hippocampus was analyzed. Only statistically significant data (*p* < 0.05; see below) or data with strong trends (*p* < 0.10; see below) is shown. To the left of each representative WB, the respective quantification of the surface/total ratio is shown. Vertical axis shows the mean fold change vs. the wildtype control (control set to a value of 1). Error bars are SEMs. *n* ≥ 5 per group. *p < 0.05, (*)*p* < 0.10.

**Figure 3:**
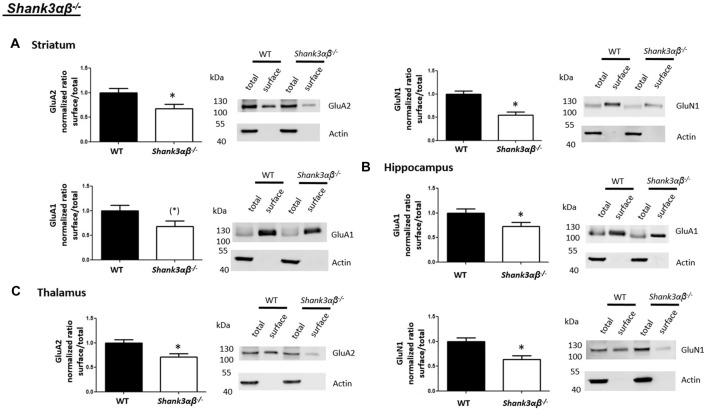
Analysis of cell surface glutamate and GABA receptor subunits in ASD related brain regions of *Shank3*αβ^−/−^ mice. **(A–C)** Representative WBs showing band intensities of total protein and cell surface sample in striatum **(A)**, hippocampus **(B)** and thalamus **(C)** of wildtype and *Shank3*αβ^−/−^ mice reveal a downregulation of cell surface glutamate receptor subunits in *Shank3*αβ^−/−^ mice. Immunodetections were carried out with antibodies directed against GABA_A_Rα1, GluA2, GluN1, GluA1, mGluR5, and actin. For GABA_A_Rα1, GluA2 and actin all brain regions were analyzed. For GluN1 all brain regions except the cerebellum were analyzed. For GluA1 all brain regions except the thalamus and cerebellum were analyzed. For mGluR5 only the hippocampus was analyzed. Only statistically significant data (*p* < 0.05; see below) or data with strong trends (*p* < 0.10; see below) is shown. To the left of each representative WB, the respective quantification of the surface/total ratio is shown. Vertical axis shows the mean fold change vs. the wildtype control (control set to a value of 1). Error bars are SEMs. *n* ≥ 5 per group. *p < 0.05, (*)*p* < 0.10.

Interestingly, we found that both *Shank2*^−/−^ and *Shank3*αβ^−/−^ mice exhibit a reduction of several cell surface glutamate receptors in the analyzed brain regions—especially in the striatum and thalamus (Figures 2, 3, 5). More precisely, *Shank3*αβ^−/−^ mice exhibit significantly reduced cell surface expression levels of GluN1 and GluA2 in the striatum and thalamus and *Shank2*^−/−^ mice have significantly reduced cell surface expression levels of GluN1 in the striatum and cortex. Furthermore, we found that *Shank3*αβ^−/−^ and *Shank2*^−/−^ mice exhibit significantly reduced cell surface expression levels of GluA1 in the hippocampus and GluA2 in the cerebellum, respectively. Additionally, several other cell surface glutamate receptors showed trends (p ≤ 0.10) for a downregulation in the analyzed brain regions (Figures 2, 3, 5). In the cortex, *Shank2*^−/−^ and *Shank3*αβ^−/−^ mice both showed no altered cell surface expression of GABA_A_Rα1, GluA2, GluA1 and *Shank3*αβ^−/−^ mice also showed no alteration of GluN1 levels. In the striatum, *Shank2*^−/−^ and *Shank3*αβ^−/−^ mice both showed no altered cell surface expression of GABA_A_Rα1 and GluA1 and *Shank2*^−/−^ mice also showed no alteration of GluA2 levels. As for the hippocampus, *Shank2*^−/−^ and *Shank3*αβ^−/−^ mice both showed no altered cell surface expression of GABA_A_Rα1, GluA2, NR 1 or mGluR5 and *Shank3*αβ^−/−^ mice also showed no alteration of GluA1 levels. For the thalamus we observed no altered cell surface expression of GABA_A_Rα1 in *Shank3*αβ^−/−^ mice and *Shank2*^−/−^ mice showed no alteration of GluA2 or GluN1 levels. Lastly, in the cerebellum *Shank2*^−/−^ and *Shank3*αβ^−/−^ mice both showed no altered cell surface expression of GABA_A_Rα1 and *Shank3*αβ^−/−^ mice showed no alteration in GluA2 levels (Figures 2, 3, 5). Of note, no differences in total expression levels of the analyzed receptors were found between *Shank2*^−/−^ or *Shank3*αβ^−/−^ mice and wildtype controls (Supplementary Figure S2,2).

**Figure 4:**
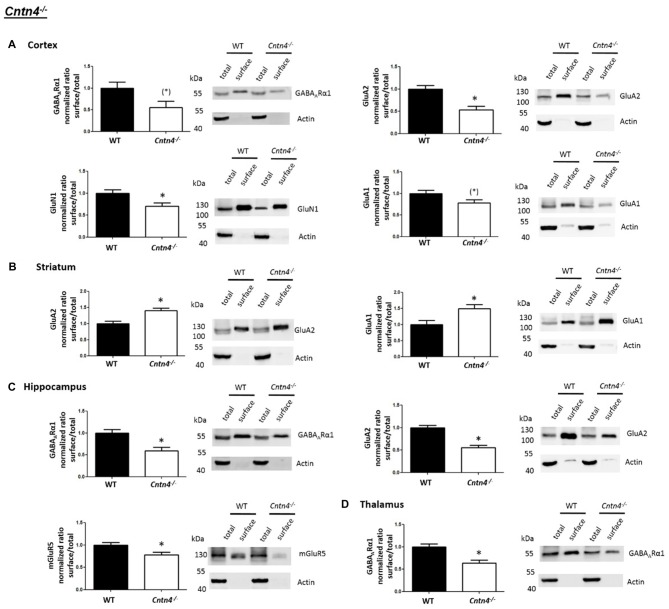
Analysis of cell surface glutamate and GABA receptor subunits in ASD related brain regions of *Cntn4*^−/−^ mice. **(A–D)** Representative WBs showing band intensities of total protein and cell surface sample in cortex **(A)**, striatum **(B)**, hippocampus **(C)** and thalamus **(D)** of wildtype and *Cntn4*^−/−^ mice reveal a dysregulation of cell surface glutamate and GABA receptor subunits in *Cntn4*^−/−^ mice. Immunodetections were carried out with antibodies directed against GABA_A_Rα1, GluA2, GluN1, GluA1, mGluR5, and actin. For GABA_A_Rα1, GluA2, and actin all brain regions were analyzed. For GluN1 all brain regions except the cerebellum were analyzed. For GluA1 all brain regions except the thalamus and cerebellum were analyzed. For mGluR5 only the hippocampus was analyzed. Only statistically significant data (*p* < 0.05; see below) or data with strong trends (*p* < 0.10; see below) is shown. To the left of each representative WB, the respective quantification of the surface/total ratio is shown. Vertical axis shows the mean fold change vs. the wildtype control (control set to a value of 1). Error bars are SEMs. *n* ≥ 5 per group. *p < 0.05, (*)*p* < 0.10.

**Figure 5:**
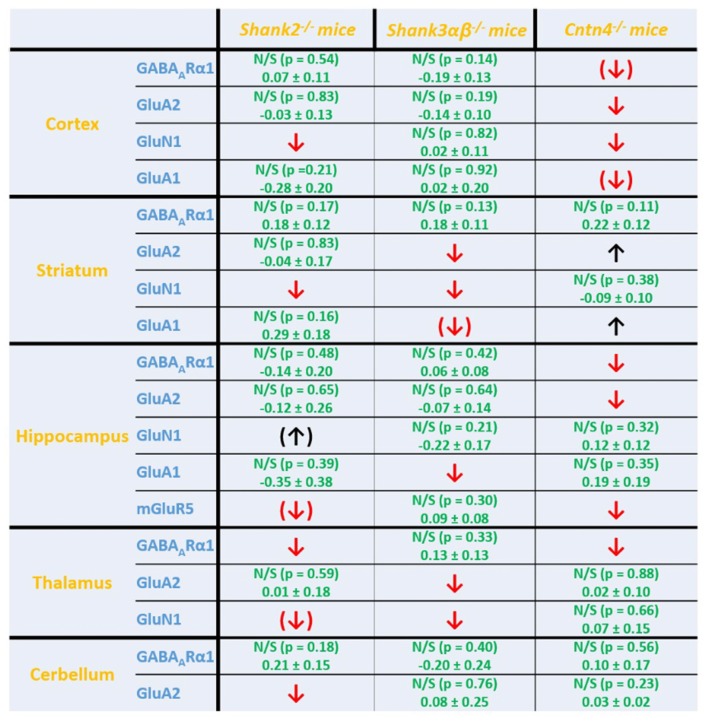
Summary of cell surface glutamate and GABA receptor subunit analysis in ASD related brain regions of *Shank2*^−/−^, *Shank3*αβ^−/−^ and *Cntn4*^−/−^ mice. Summarized cell surface glutamate and GABA receptor subunit analysis reveals a commonality of *Shank2*^−/−^ and *Shank3*αβ^−/−^ mice with respect to dysregulations of cell surface receptors relative to wildtype controls whereas *Cntn4*^−/−^ mice appear to differ from the *Shank* deficient mice in this respect. On the whole, *Shank2*^−/−^ and *Shank3*αβ^−/−^ mice display reduced levels of cell surface glutamate receptor subunits in most of the analyzed brain regions, whereas dysregulation of cell surface GABA_A_Rα1 levels is minimal. In contrast, *Cntn4*^−/−^ mice display dysregulations of both cell surface glutamate and GABA receptor subunits. Summary is based on quantifications of surface/total ratio (Figures 2–4). ↓, ↑ *p* < 0.05; (↓), (↑) *p* < 0.10. In the case of non-significant data (N/S) which does not show trends (*p* > 0.10) the difference of means (wildtype—autism mouse model) ± SEM and the corresponding *p*-value is shown. Numbers are rounded to two decimal places. Upregulated (↑); downregulated (↓).

### *Cntn4*^−/−^ Mice Exhibit Altered Cell Surface Glutamate and GABA Receptor Levels in ASD Related Brain Regions

We then expanded our investigation of cell surface glutamate and GABA receptors in ASD mouse models by applying the cell surface biotinylation protocol to acute slices from *Cntn4*^−/−^ mice and comparing them to wildtype controls. Again, western blotting was carried out to analyze samples from the cortex, striatum, hippocampus, thalamus and cerebellum (Supplementary Figure S1).

In contrast to the findings of *Shank2*^−/−^ and *Shank3*αβ^−/−^ mice, we found that *Cntn4*^−/−^ mice exhibit reduced cell surface glutamate receptor levels in the cortex and hippocampus but increased cell surface glutamate receptor levels in the striatum when compared to wildtype controls (Figures 4, 5). More precisely, *Cntn4*^−/−^ mice exhibit significantly reduced cell surface expression levels of GluA2 and GluN1 in the cortex, reduced cell surface expression levels of GluA2 and mGluR5 in the hippocampus, and increased cell surface expression levels of GluA2 and GluA1 in the striatum. In addition to these dysregulations of cell surface glutamate receptor levels we also found changes in cell surface GABA receptor levels in several brain regions (Figures 4, 5). More precisely, we found reduced cell surface GABA_A_Rα1 levels in the hippocampus and thalamus and a trend for reduced cell surface GABA_A_Rα1 levels in the cortex. In contrast, except for reduced thalamic cell surface GABA_A_Rα1 levels in *Shank2*^−/−^ mice (Figures 2, 5), no further differences between *Shank2*^−/−^ or *Shank3*αβ^−/−^ and wildtype mice could be identified with regard to cell surface GABA_A_Rα1 levels. In the cortex, *Cntn4*^−/−^ mice displayed no significantly altered cell surface expression of GABA_A_Rα1 and GluA1 and in the striatum GABA_A_Rα1 and GluN1 levels were not altered. As for the hippocampus, *Cntn4*^−/−^ mice displayed no significantly altered cell surface expression of GluN1 and GluA1 and in the thalamus *Cntn4*^−/−^ mice displayed no changes in GluA2 and GluN1 levels. Lastly, in the cerebellum *Cntn4*^−/−^ mice displayed no significantly altered cell surface expression of GABA_A_Rα1 and GluA2 (Figures 4, 5). Of note, no differences in total expression levels of the analyzed receptors were found between *Cntn4*^−/−^ mice and wildtype controls (Supplementary Figure S2,2).

## Discussion

Epidemiological studies suggest that up to 1% of the world’s population may be diagnosed with ASD (Elsabbagh et al., 2012; Christensen et al., 2016) and, therefore, understanding the genetic/molecular underpinnings of this class of psychiatric disorders is of great importance. Years of research have generated data which clearly suggests a strong genetic component involved in the etiology of ASDs—e.g., variations in the *SHANK2*, *SHANK3* and *CNTN4* genes have been associated with an increased risk for developing ASD and many knockout mice of these genes display abnormalities in behavior and social interactions reminiscent of ASD patients (Leblond et al., 2012; Provenzano et al., 2012; Schmeisser et al., 2012; Jiang and Ehlers, 2013). Nonetheless, the scientific community lacks a unifying theory backed up by experimental data to explain how a plethora of genetic variations leads to a condition with similar core symptoms, albeit with a great heterogeneity and variability (Mullins et al., 2016).

**Figure 6:**
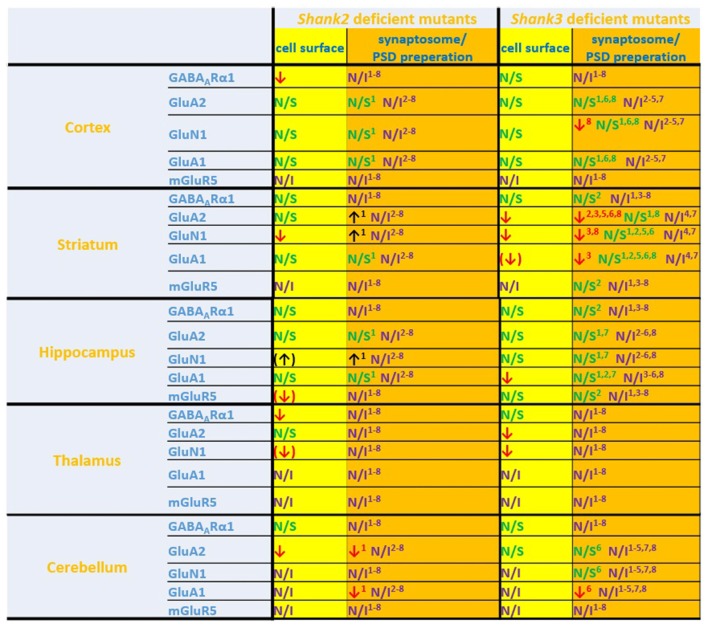
Comparison of cell surface glutamate receptor subunit analysis with synaptosome/postsynaptic density (PSD) preparation analysis of glutamate receptors in ASD related brain regions in *Shank2* and *Shank3* deficient mice. Cell surface glutamate receptor subunit analysis and synaptosome/PSD preparation analysis reveals that the results of both methods do not necessarily show similar trends but both approaches usually generate converging data. ^1^Refers to data from Schmeisser et al. (2012). ^2^Refers to data from Peca et al. (2011). ^3^Refers to data from Reim et al. (2017). ^4^Refers to data from Peter et al. (2016). ^5^Refers to data from Jaramillo et al. (2016). ^6^Refers to data from Mei et al. (2016). ^7^Refers to data from Kouser et al. (2013). ^8^Refers to data from Zhou et al. (2016); note that in this study two different and *Shank3* deficient mice were analyzed. Cell surface refers to data from the current work: ↓ *p* < 0.05; (↓), (↑) *p* < 0.10. N/I stands for not investigated and N/S stands for non-significant results. Figure only shows data from brain regions/antibody detections which were addressed in this study. Upregulated ↑; downregulated ↓.

In this study, we compared cell surface expression levels of several glutamate and GABA receptors in ASD related brain region of three different ASD mouse models (*Shank2*^−/−^, *Shank3*αβ^−/−^ and *Cntn4*^−/−^) aiming on unraveling molecular abnormalities in excitatory and/or inhibitory signaling components which are common to all three mouse models. It is important to note that the mouse models used in this study differ with respect to their ASD-related behavior: *Shank2*^−/−^ and *Shank3*αβ^−/−^ mice clearly show repetitive, stereotyped behavior and deficits in social interactions, though in varying degrees (Schmeisser et al., 2012; Vicidomini et al., 2017; Monteiro and Feng, 2017), whereas ASD-related behavior in *Cntn4*^−/−^ has been less well characterized but appears to be much less pronounced (Molenhuis et al., 2016). Our data suggest that—as might have been expected—genetic deficiency of *Shank2* and *Shank3* yields relatively similar changes in cell surface glutamate and GABA receptor expression levels: on the whole, a downregulation of cell surface ionotropic glutamate receptor subunits (GluN1, GluA2 and GluA1) could be observed in several ASD-related brain regions (especially in the striatum and thalamus) whereas almost no changes in the expression of cell surface ionotropic GABA receptors was seen. Since ionotropic receptors at the cell surface are well-positioned to take part in neuronal signaling at the synapse, our data suggests a reduction in the excitation/inhibition (E/I) ratio in several brain regions of *Shank2*^−/−^ and *Shank3*αβ^−/−^ mice. In line with this hypothesis, *Shank3* deficient mice have been reported to have a reduced striatal/cortico-striatal glutamatergic synaptic transmission and a reduced spine density in the striatum (Peca et al., 2011). Additionally, several *Shank2* and *Shank3* deficiency mouse models display a reduced glutamatergic synaptic transmission and a reduced spine density/PSD ultrastructure in the hippocampus (Schmeisser et al., 2012; Won et al., 2012; Jiang and Ehlers, 2013). Unfortunately, to our knowledge there is no published electrophysiological or electron microscopy data of the thalamus available for *Shank* deficient mice but it would be intriguing to find out whether thalamic abnormalities in glutamatergic signaling could be identified. In the case of *Cntn4*^−/−^ mice we also found abnormalities in cell surface receptors. However, we observed that these mice exhibit both brain region specific down- and upregulations of cell surface glutamate receptors and brain region specific downregulations of GABAergic cell surface receptors. Essential differences between Cntn4 and Shanks may underlie these differential changes observed in our mouse models. In particular, the expression of Cntn4 in the brain is far lower and more restricted than that of the Shanks. Overall, Cntn4 expression is low in the brain with a few hotspots such as several thalamic nuclei, hippocampal CA regions and several cortical cell-types, including pyramidal as well as GABAergic neurons (Habib et al., 2016; Tasic et al., 2016; Oguro-Ando et al., 2017). This expression pattern may be related to the changes in cell-surface receptor expression levels observed in the *Cntn4*^−/−^ mice.

The findings of this study point towards the possibility that there may not be a common molecular phenotype that all ASD mouse models share. On the one hand, it suggests that other levels of molecular and cellular processes may be a common point of convergence. On the other hand, it indicates that gene-specific effects may diversify phenotypes as is also observed in individuals with ASD who display great heterogeneity in symptoms, severity, and comorbidities. Therefore, future treatment of ASD patients might have to be highly individualized.

Of note, our study differs from virtually all previous biochemical studies of synaptic proteins in ASD mouse models since we implemented a biotinylation assay to analyze cell surface bound receptors as opposed to the commonly used analysis of synaptosomal/PSD fractions. Obviously, the advantage of analyzing cell surface receptors is that the data has more implications for synaptic signal transmission, since synaptosomal/PSD fractions will contain many receptors, which cannot participate in synaptic signaling, as they are not localized at the cell surface (e.g., localization in the spine before exocytosis or after endocytosis). This is of particular relevance for the research in the field of ASD since it is likely that the common abnormality that all forms of ASD share might be alterations at the level of neuronal signaling/neuronal networks (Bourgeron, 2015; Mullins et al., 2016). The reader should, however, keep in mind that the biochemical experimental approach of our study does not allow a discrimination of neuronal subtypes, extrasynaptic and synaptic receptors or neurons and glia cells. And since e.g., astrocytes have been shown to express glutamate receptors (Spreafico et al., 1994; Seifert et al., 1997; Gallo and Ghiani, 2000; Verkhratsky and Kirchhoff, 2007) they, too, should be kept in mind when interpreting our data in terms of neuronal/network signaling and changes in the E/I balance (Hansson and Rönnbäck, 2003; Araque and Navarrete, 2010). In line with this, *Shank2* deficiency in defined neuronal cell types appears to change E/I (Kim et al., 2018), suggesting that neuron-type-specific effects of Shank deletions must be addressed in future work. Nonetheless, one should also bear in mind in this context that the striatum is somewhat of an exception since around 95% of striatal neurons are GABAergic medium sized spiny neurons (Yager et al., 2015). So in the case of the striatum our data may, indeed, represent receptor levels of a rather restricted number of neural cells and probably reflects receptor levels of medium sized spiny neurons to a large degree. Lastly, future work on ASD mouse models would greatly benefit from an experimental design which can distinguish between extrasynaptic and synaptic receptors since, e.g., extrasynaptic GABA_A_ receptors have been shown to modulate epileptic seizures in mouse models by affecting tonic inhibition (Glykys and Mody, 2006; Heise et al., 2017) and it is known that ASD patients have an increased risk for developing epileptic seizures (Besag, 2018).

If one compares the data of this study with the available data of brain region specific synaptosome/PSD fraction analysis we find that in many cases the trends are similar, in others they are not (Figure 6). Thus, this study is also a cautionary note that one should be careful when inferring properties of synaptic signaling from synaptosomal/PSD fraction data. The best approach would be to combine synaptosome/PSD fraction analysis with biotinylation assays to get a more complete overview of the proteome at the synapse.

## Author Contributions

CH and JP carried out the majority of experiments and data analysis. Also, JS, CB, JK and RS helped in carrying out some of the experiments of this work. CH established the experimental design in the laboratory, carried out the literature research, wrote the manuscript and created the figures. MKas and JPB provided mice for the Cntn4 experiments and provided input to the manuscript. MK and TB designed the experiments, revised the text and provided expertise for the project.

## Conflict of Interest Statement

The authors declare that the research was conducted in the absence of any commercial or financial relationships that could be construed as a potential conflict of interest.

## Abbreviations

ACSF: artificial cerebrospinal fluid
ASD: autism spectrum disorders
d: distilled
E/I: excitation/inhibition
kDa: kilo Dalton
LTP: long-term potentiation
PBS: phosphate buffered saline
PSD: postsynaptic density
RPM: rotations per minute
SDS: sodium dodecyl sulfate
SDS-PAGE: sodium dodecyl sulfate polyacrylamide gel electrophoresis
Shank: SH3 and multiple ankyrin repeat domains 3, also known as ProSAP.
