# Supplementary material for: Heterogeneity of Cell Surface Glutamate and GABA Receptor Expression in Shank and CNTN4 Autism Mouse Models
Source: Front Mol Neurosci. 2018 Jun 19;11:212. doi: 10.3389/fnmol.2018.00212 (PMC6018460; doi:10.3389/fnmol.2018.00212)
Supplement: Supplementary file 1 [file Data_Sheet_1.docx]

Supplementary Material

# Heterogeneity of cell surface glutamate and GABA receptor expression in Shank and CNTN4 autism mouse models

Christopher Heise^1,2^, Jonathan M. Preuss^1^, Jan C. Schroeder^1^, Chiara R. Battaglia^1^, Jonas Kolibius^1^, Rebecca Schmid^1^, Michael R. Kreutz^2^, Martien J.H. Kas^3,4^, J. Peter H. Burbach^4^, and Tobias M. Boeckers^1*^

**^*^ Correspondence:** Tobias M. Boeckers, Institute of Anatomy and Cell Biology, Ulm University, Albert-Einstein-Allee 11, Ulm, 89073, Germany, tobias.boeckers@uni-ulm.de

**Supplementary Figures**


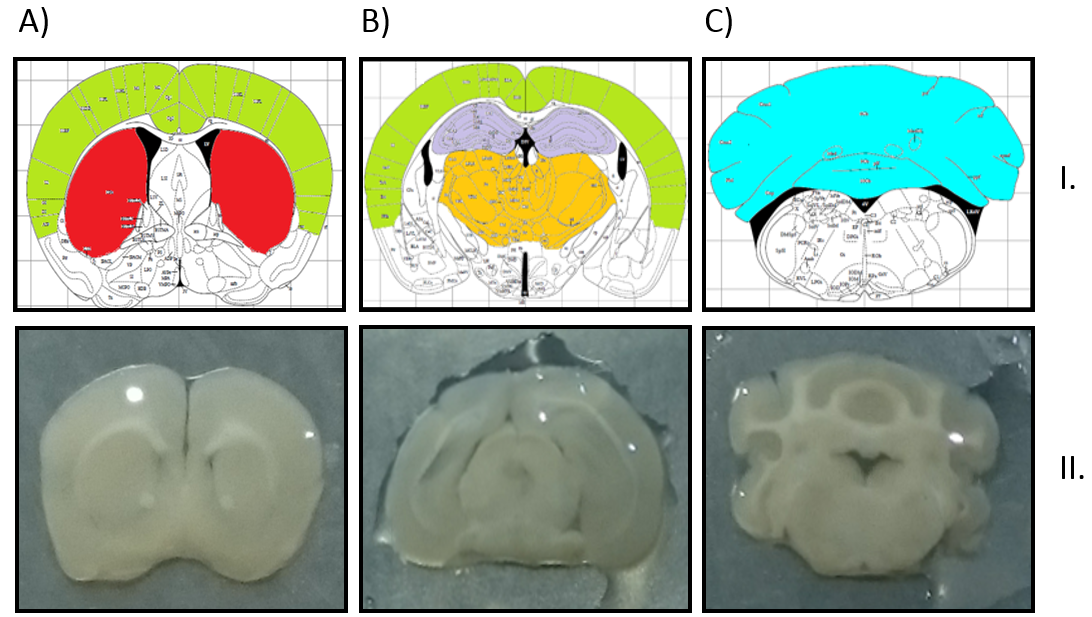


**Supplementary Figure S1: Visual impression of coronal slices resulting from slice preparation**

**I,** Coronal sections according to Mouse-Brain-Atlas-Paxinos (2001), color coding superimposed. **A)** Slices containing striatum (red) and anterior cortex potrions (green) were gathered between Bregma 1.50 mm- Bregma 0.00 mm. **B)** Slices containing hippocampus (grey), thalamus (yellow), and posterior cortex portions (green) were gathered between Bregma -1.00 mm and Bregma -3.00 mm. **C)** Slices containing cerebellum (turquoise) were gathered between Bregma -5.80 mm and Bregma -7.80 mm. **II,** corresponding native coronal slices (thickness: 300µm).

**
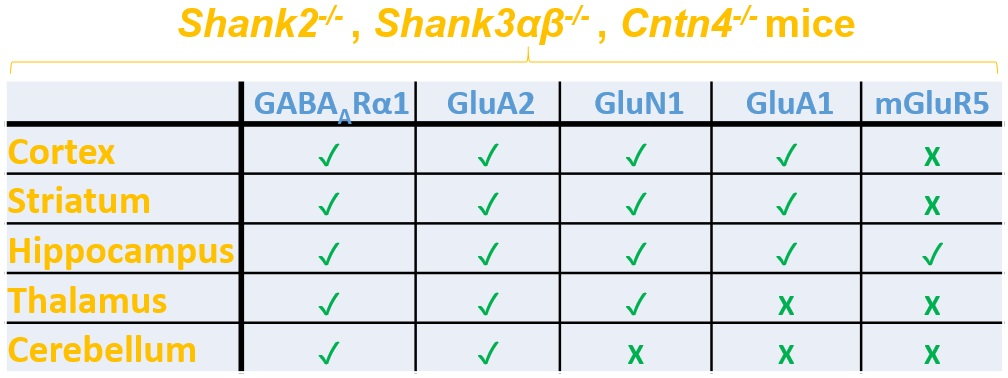
**

**Supplementary Figure S2,1: Signal intensity analysis of cell surface glutamate and GABA receptor subunits in ASD related brain regions of *Shank2^-/-^*, *Shank3αβ^-/-^*, and *Cntn4^-/-^* mice**

Signal intensity of cell surface glutamate and GABA receptor subunits in ASD related brain regions of *Shank2^-/-^*, *Shank3αβ^-/-^*, and *Cntn4^-/-^* mice was sufficient for quantifying antibodies in most of the analyzed brain regions. Signal intensity of cell surface glutamate and GABA receptors was assessed by western blot. X indicates that signal intensity was insufficient for quantification, ✓ indicates sufficient signal intensity and n ≥ 5 (**Figures 2-5**).


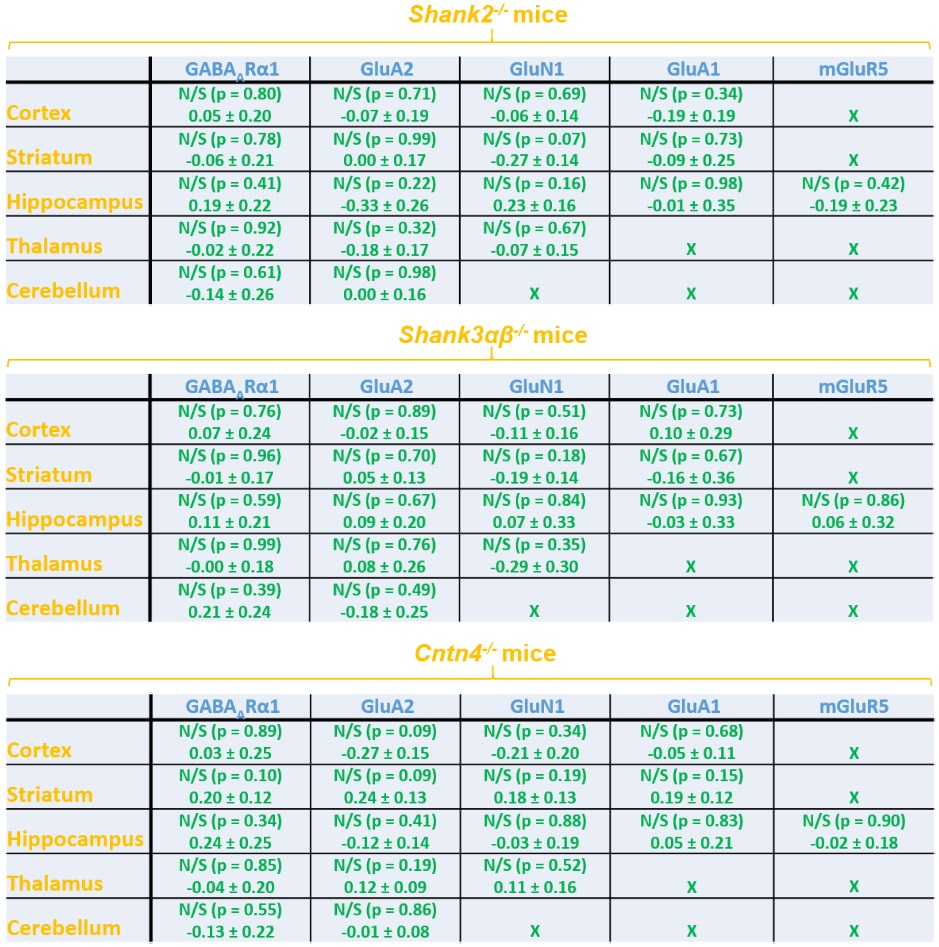


**Supplementary Figure S2,2: Analysis of total glutamate and GABA receptor subunits levels in ASD related brain regions of *Shank2^-/-^* mice, *Shank3αβ^-/-^*, and *Cntn4^-/-^* mice**

Immunodetections were carried out with antibodies directed against GABAARα1, GluA2, GluN1, GluA1, mGluR5, and actin. For GABAARα1, GluA2, and actin all brain regions were analyzed. For GluN1 all brain regions except the cerbellum were analyzed. For GluA1 all brain regions except the thalamus and cerbellum were analyzed. For mGluR5 only the hippocampus was analyzed. Data was normalized to wildtype and set arbitrarily to 1 (view materials and methods for more details). N/S stands for non-significant results. Mean difference (wildtype – autism mouse model) +/- SEM and the corresponding p-value is shown. Numbers are rounded to two decimal places.
